# Monobutyrin and Monovalerin Affect Brain Short-Chain Fatty Acid Profiles and Tight-Junction Protein Expression in ApoE-Knockout Rats Fed High-Fat Diets

**DOI:** 10.3390/nu12041202

**Published:** 2020-04-24

**Authors:** Thao Duy Nguyen, Frida Fåk Hållenius, Xue Lin, Margareta Nyman, Olena Prykhodko

**Affiliations:** Department of Food Technology, Engineering and Nutrition, Kemicentrum, Lund University, PO Box 124, SE-221 00 Lund, Sweden

**Keywords:** SCFAs, cholesterol, butyric acid, gut–brain axis, valeric acid, isovaleric acid

## Abstract

Monobutyrin (MB) and monovalerin (MV), esters of short-chain fatty acids (SCFAs), have previously been shown to reduce liver cholesterol and inflammation in conventional rats fed high-fat diets. This study explored the potential effects of MB and MV in hypercholesterolemic apolipoprotein E-knockout (ApoE-/-) rats. ApoE-/- rats were fed three high-fat (HF) diets, pure or supplemented with MB or MV (1%), for 5 weeks. One group of conventional rats (C) was also fed the pure high-fat diet and another group of ApoE-/- rats a low-fat (LF) diet. Blood and liver lipids, urinary lactulose/mannitol, SCFAs (blood and brain), tight junction proteins (small intestine and brain), and inflammation-related markers (blood, brain, and liver) were analyzed. MV supplementation elevated serum high-density lipoprotein (HDL) cholesterol and valeric acid concentration (*p* < 0.05), while the amounts of isovaleric acid in the brain were reduced (*p* < 0.05). MB increased butyric acid amounts in the brain, while the plasma concentration of interleukin 10 (IL-10) was lowered (*p* < 0.05). Both MV and MB upregulated the expression of occludin and zonula occludens-1 (ZO-1) in the brain (*p* < 0.05). Supplementation of MB or MV affected HDL cholesterol, the expression of tight junction proteins, and SCFA profiles. MB and MV may therefore be promising supplements to attenuate lipid metabolic disorders caused by high-fat intake and genetic deficiency.

## 1. Introduction

Increasing scientific data support the harmful effects of an animal-derived high-fat diet in the development of several metabolic diseases [1,2,3]. High-fat diets stimulate disturbances in lipid metabolism and activity of the gastrointestinal microbial community, leading to an impaired gut barrier function [4]. When the permeability of the intestinal epithelium increases, bacterial components, such as lipopolysaccharide (LPS) and other potentially harmful substances, including toxins, may enter the circulation, causing systemic inflammation. These events are common hallmarks of lipid and inflammation-related disorders, such as obesity.

Dietary fiber may have a protective effect against diet-induced obesity. Despite being a minor component, with a recommended intake of about 25 to 30g/day (3g/MJ) for a human adult, adding fiber to a high-fat (high-calorie) diet has been shown to decrease inflammation and improve glucose homeostasis [2]. Mechanisms responsible for the protective effects of dietary fiber are inconclusive, but the end-products generated by microbial degradation of dietary fiber in the colon have attracted attention. Among these metabolites, short-chain fatty acids (SCFAs) have emerged as promising candidates due to their universal actions, from providing energy to the mucosal cells in the colon to modulating immune responses and gene expression. Levels of SCFAs generally increase at the actual site of fermentation and in the circulation following the consumption of fiber-rich diets. The protective effects of SCFAs in the colon, especially butyric acid, are associated with an improved barrier function by upregulating tight junction protein expressions [5]; reducing the translocation of bacterial components, such as LPS, to the blood circulation [6]; and reducing cholesterol absorption [7]. SCFAs can have a modulating impact on other peripheral organs like the liver, heart, spleen, and pancreas, and even the brain, when reaching the blood stream by means of transporters or receptors [8].

Apolipoprotein E (ApoE) is an essential cholesterol-rich lipoprotein transporter, expressed in several cells in the peripheral system (hepatocytes, adipocytes, and macrophages) and in the brain (mainly by astrocytes and under stressed conditions by neurons) [9]. ApoE is associated with very low-density lipoprotein (VLDL) particles in plasma, and with high-density lipoprotein cholesterol (HDL)-like particles in the brain. ApoE deficiency leads to hyperlipidemia, characterized by comparatively low blood levels of HDL cholesterol (HDL-c) and high levels of total and low-density cholesterol (LDL-c). In humans, variants of the ApoE gene, i.e., the ApoE ε4 allele, increase the risk of developing Alzheimer’s disease (AD), possibly due to its association with cardiovascular disease [10]. Elevated plasma cholesterol and absence of the ApoE gene have been shown to contribute to disturbances of the blood–brain barrier (BBB) in mice [11].

ApoE knockout (ApoE-/-) animal models provide valuable means for studying and exploring potential targets to suppress the burden of disturbed lipid metabolism-related diseases, such as obesity, cardiovascular diseases, and AD. In this respect, rats are less commonly used than mice for generating transgenic animals, since it is more difficult to inject DNA into fertilized ova [12], but with the advanced and rapid development of current gene-editing technologies, more transgenic rat models are available. Rats also have advantages, such as a larger volume of blood and tissue collection, thus allowing more analyses compared to mice.

We and other groups have previously reported that supplementation of SCFA esters in high-fat diets and given to conventional rats and mice can suppress lipid-induced disturbances, improve the intestinal barrier function, and reduce inflammation and gut dysbiosis [13,14,15,16]. Inspired by these findings, the present study was carried out to investigate whether the glycerol esters, monobutyrin (MB) and monovalerin (MV), supplemented to feed in a dose of 1%, could have any effects in high-fat-fed rats, where the ApoE gene has been knocked out. High-fat diets and ApoE deficiency are also suggested to impair the gut–brain barrier function involved in the onset of obesity and neuropathological disorders, including AD [3,11,17,18,19]. Therefore, we explored the effects of MB and MV by assessing the lipid profile, SCFA levels and their receptors, gut–brain barrier function (intestinal permeability, gene expression of tight junctions in the brain and small intestine, and mucosal thickness), and some markers usually associated with inflammation.

## 2. Materials and Methods 

### 2.1. Ethical Permission

The use and treatment of animals in this study were evaluated and permission granted by the Local Ethical Review Committee for animal research at Lund University, with the approval number M114-15.

### 2.2. Animals and Diets

A total of 50 male rats (40 ApoE-/- and 10 conventional rats, 4–8 weeks old) with the same genetic background of Sprague-Dawley strain were shipped from SAGE Lab, Inc. (Boyertown, USA) [20]. On arrival, the rats were adapted to the animal house conditions for 4 days, before entering the 5-week dietary intervention. The ApoE-/- rats were randomly divided into four groups (*n* = 10/group), based on a similar body weight. Each group was housed in different cages, with 2–3 rats per cage. Three groups were given high-fat diets with a very similar content of nutrients (Table S1), i.e., a pure high-fat diet (HF, without any supplementation of MV and MB) or with supplementation of monobutyrin (MB) or monovalerin (MV) (10 g/kg diet, dry weight basis). The fourth group of ApoE-/- rats was also given a pure diet but with a low-fat content (LF). One group of conventional rats (C) given the pure high-fat diet was also included in the experiment to be able to compare effects between ApoE-/- rats and conventional rats. The dose of MB and MV chosen was based on our previous results [14,15,16]. Lard was used as the only fat source in the high-fat diet regimen, while rapeseed oil was the fat source in the low-fat diet. Animals had free access to water and experimental diets throughout the study period. The amount of feed given to the rats in each cage was registered as well as feed residues.

### 2.3. Experimental Design

Feed intake and body weight gain were recorded weekly during the five-week study. Changes in blood lipids from the tail vein were measured twice a week. After three weeks on the test diets, the rats were placed individually in metabolic cages for three days to perform in vivo intestinal permeability tests, after which the rats were returned to their housed cages. At the end of the experiment, the rats were euthanized by a subcutaneous injection mixture of Hypnorm (Division of Janssen-Cilag Limited, Janssen Pharmaceutica), Dormicum (Accord Healthcare, London, UK), and autoclaved Millipore water in proportion (1:1:2), at a volume of 0.15 mL/100 g body weight. Blood was collected from the portal vein and aorta, centrifuged, and serum and plasma were separated using corresponding blood collection tubes from Becton Dickinson BD Vacutainer® SST™ (Franklin Lakes, USA), and saved at −40 °C for analyses of the lipids, SCFAs, cytokines, and liver enzymes. White blood cells were counted in whole blood samples mixed with Türk’s solution in a 1:20 proportion immediately after blood collection using a Bürker chamber. Glucose in the portal vein serum was determined with a HemoCue^®^ Glucose 201+ Analyzer from HemoCue AB (Ängelholm, Sweden). Liver, spleen, small intestine (divided into three parts, i.e., duodenum, jejunum, and ileum), caecum, colon, and brain were collected and stored at −80 °C for further analysis.

### 2.4. Analyses

#### 2.4.1. Lipids

Total cholesterol, LDL cholesterol, HDL cholesterol, and triglycerides (TG) were measured in the tail vein, portal vein serum, and aortic plasma, using commercial kits from Thermo Fisher Scientific Inc. (Middletown, OH, USA). Total cholesterol and TG in the liver were analyzed, following a low-toxic lipid extraction procedure [21], as previously applied [16].

#### 2.4.2. Intestinal In Vivo Permeability

The intestinal in vivo permeability test in rats was conducted by measuring the absorption of lactulose and mannitol according to Meddings and Gibbons [22,23], as previously described [16]. Concentrations of lactulose and mannitol quantitatively collected in urine were analyzed with the EnzyChromTM Intestinal Permeability Assay Kit (EIPM-100) from BioAssay System (Hayward, CA, USA).

#### 2.4.3. Mucosal Thickness

The mucosal thickness was measured in three parts of the small intestine, including the duodenum, middle part of the jejunum and ileum, and in the upper colon of the rats according to previous procedures with some modifications [23,24]. After fixation in 4% paraformaldehyde, all tissue samples were washed with phosphate-buffered saline and cryoprotected with 30% sucrose by incubation for 24 h. All tissues were embedded into cryo-moulds (Sakura, Torrance, CA, USA) using the optimal cutting temperature compound TissueTek® (HistoLab, Gothenburg, Sweden), immediately frozen on dry ice, and stored at −80 °C. The cryosection of animal tissues was performed on a Leica CM1860 Cryostat (Leica Microsystems AB, Germany). Sections, 6-μm thick, were mounted on polylysine-coated adhesion slides (ThermoFisher Scientific), dried for one hour at room temperature, and stored at −20 °C. Hematoxylin and eosin (H&E) staining was then performed according to the standard protocol and tissues were mounted under a coverslip and examined under an Olympus microscope BX60 (Olympus, Tokyo, Japan). Images of tissues selected for morphometric analyses were taken by an Olympus DP74 camera using a lens with x4 magnification and CellSens Entry imaging software. Mucosal thickness, from the top of the villi to the muscularis mucosa, was measured using the ImageJ open program (NIH in Bethesda, Maryland, USA), with 15–20 repetitions per tissue sample.

#### 2.4.4. SCFAs

A gas-chromatographic method was used to measure serum SCFA concentrations, where the SCFAs were pre-enriched and extracted with hollow fiber [25]. Brain samples were homogenized with NaCl 0.9% solution (ratio 1:3, weight/volume) on ice, and the suspension was then centrifuged at 15,000 rpm (or 25,000× *g*) for 20 min at 4 °C prior to hollow fiber extraction from the collected supernatants.

#### 2.4.5. Gene Expression in Small Intestine and Brain

Approximately 20 (small intestine) or 40 mg (brain) of tissue was used to extract total RNA with on-column DNA digestion, using the RNeasy Mini Kit or RNeasy Lipid Tissue Mini Kit, respectively, according to the manufacturer’s instructions (Qiagen Inc.). RNA purity and concentration were checked with the Qubit® 4.0 Fluorometer (Thermo Fisher Scientific Inc.). Next, 500 ng of total RNA was used to synthesize cDNA using the Thermo ScientificTM RevertAidTM First Strand cDNA Synthesis Kit (Thermo Fisher Scientific Inc.). Finally, cDNA at a concentration of 5 ng/µL was amplified with quantitative polymerase chain reaction using a previously described protocol [15]. Predesigned primers for rat occludin (gene *Ocln*), zonula occludens-1/ZO-1 (*Tjp1*), G-protein-coupled receptor 43/GPR43 (*Ffar2*), GPR109A (*Nlacr1*), and ribosomal protein (*Rpl13a*) were purchased from Sigma-Aldrich (St. Louis, MO, USA). Relative mRNA expression was calculated according to the ΔΔCt method, using *Rpl13a* as an internal control.

#### 2.4.6. Cytokines in Blood and Brain and Liver Enzymes

IL-1β and IL-10 in blood plasma were measured by commercial sandwich immunoassay kits purchased from R&D Systems, Inc. (Minneapolis, USA), while alanine transaminase activity (ALT) in portal vein serum was detected by a colorimetric enzymatic kit from Abcam (Cambridge, UK). Prior to determination of IL-1β in the brain, the tissues of 350–400 mg were homogenized in five pre-chilled volumes of 25 mM Tris-HCl buffer, pH 7.4, containing 1 mM ethylenediaminetetraacetic acid (EDTA), 2 mM dithiothreitol (DTT), and a cocktail of protease inhibitors (Roche Molecular Systems Inc., Pleasanton, CA, USA). The homogenates were centrifuged at 20,000× *g* for 60 min at 4 °C and supernatant was collected for analysis.

### 2.5. Statistical Analyses and Calculations

Data are presented as means and standard errors of the means (SEM) and were evaluated using GraphPad Prism (version 8). ApoE-/- rats fed the pure high-fat diet (HF) were compared with conventional rats (C) fed the same high-fat diet and with ApoE-/- rats fed the low-fat diet (LF). Within ApoE-/- rats, the supplemented groups (MB and MV) were compared with the non-supplemented groups (either the HF or LF group). Where applicable, differences between the MB or MV group with the C group were also reported. The normality of the data was checked with the D’Agostino and Pearson test, while outliers were identified with the ROUT (combining robust regression and outlier removal) test. For comparisons of more than three groups, one-way ANOVA was used, followed by post-hoc tests, either Dunnett’s or Dunn’s for parametric or nonparametric data, respectively. For two-group comparisons, the unpaired t-test or Mann–Whitney test was applied. The Spearman correlation test was used to find connections between different variables. Projection to Latent Structures-Discriminant Analysis (PLS-DA) in SIMCA software (version 15, Umetrics, Umeå, Sweden) was applied to get a visualization of whether changes in the measured metabolites could be influenced by the experimental diets. Significant differences were *p* < 0.05. Tendency was defined as 0.05 ≤ *p* ≤ 0.1.

SCFAs in the brain are presented as total amounts (pools), i.e., by multiplying the analyzed concentrations in the brain with its weight. The same calculation was applied for liver lipids.

All assays were performed with *n* = 10/group, except the gene expression, SCFAs, and IL-1β in the brain, with *n* = 8/group, due to the limited amounts of samples. 

## 3. Results

### 3.1. Effects of Monobutyrin and Monovalerin on Serum and Liver Lipids 

ApoE-/- rats given the pure high-fat diet (HF) had significantly higher concentrations of lipids (*p* < 0.01–*p* < 0.0001) in the liver as well as in the tail (both at 2 and 4 weeks) and portal vein (at 5 weeks, i.e., upon completion of the trial) than the corresponding parts in conventional rats (C) given the same high-fat diet (Table 1). The levels were also significantly (*p* < 0.01–*p* < 0.0001) higher than in rats fed a low-fat diet (LF).

Of the different high-fat ApoE-/- groups, MV had an increased mean value of the portal serum HDL cholesterol concentration compared with the HF group (Table 1, *p* < 0.05). No further effects of MB and MV on the mean values of serum and liver lipids were found. However, when studying individual rats between weeks 2 and 4, some rats in groups fed MB, MV, and LF had significantly lower (*p* < 0.05) TG values over time, which could not be seen in the HF group (Figure S1a–d). In the MB group, there was a decrease for 8 of 10 rats, from 4.0 ± 0.3 to 3.2 ± 0.2 after 4 weeks (*p* = 0.0133). With the MV and LF groups, 6 of the 10 rats showed lower values after 4 weeks: 4.5 ± 0.3 vs. 3.1 ± 0.2 (*p* = 0.0313) and 2.2 ± 0.1 vs. 1.9 ± 0.1 (*p* = 0.013), respectively. The decrease was significantly greater in the MV group than in the LF group (30% vs. 17%, *p* = 0.004).

### 3.2. Effects of Monobutyrin and Monovalerin on Intestinal Permeability and Mucosal Thickness

**Figure 1:**
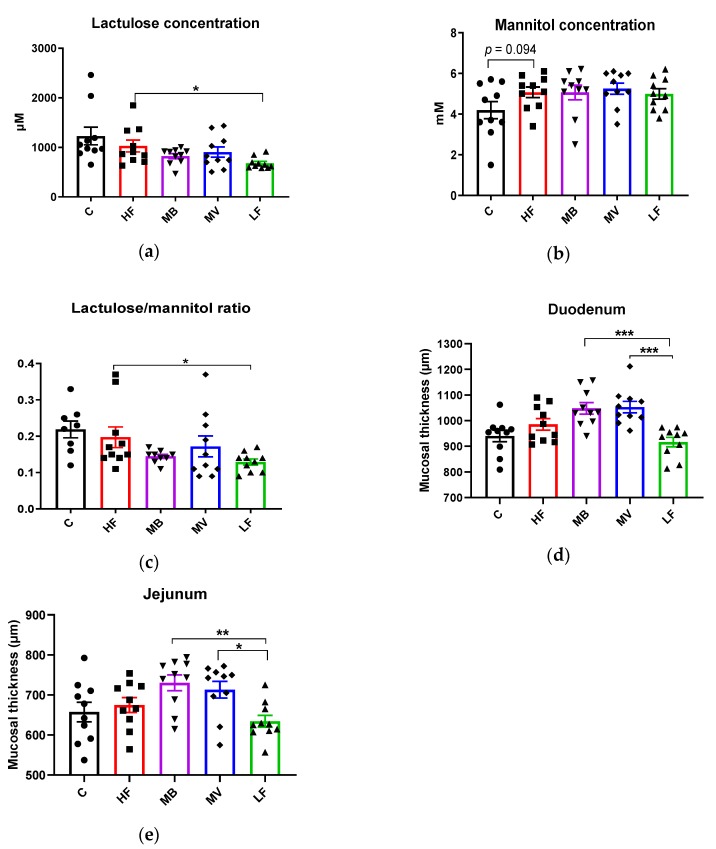
Changes in (**a**) lactulose (µM), (**b**) mannitol (mM), and (**c**) lactulose/mannitol in urine, and mucosal thickness (µm) in the (**d**) duodenum and (**e**) jejunum of conventional rats fed a pure high-fat diet (C), or Apolipoprotein E-knockout (ApoE-/-) rats fed a low-fat diet (LF) or high-fat diet (HF), pure or supplemented with 1% monobutyrin (MB) or monovalerin (MV) for 5 weeks (*n* = 10/group). Rats from the C, HF, MB, MV and LF groups are shown as ●, ■, ▼, ♦ and ▲, respectively. Values are means ± SEM. * *p* < 0.05, ** *p* < 0.01.

ApoE-/- rats fed the pure high-fat diet (HF) had a higher (Figure 1a, *p* < 0.05) excretion of lactulose in urine than ApoE-/- rats given the low-fat diet (LF), while no significant differences could be seen for mannitol. The HF group tended to have higher urinary mannitol than the C group (Figure 1b, *p* = 0.094). Inclusion of MB in the high-fat diet lowered the concentrations of lactulose excreted in the urine but not significantly (Figure 1a, *p* = 0.13). The mannitol concentration was unaffected.

The ratio lactulose/mannitol was significantly (Figure 1c, *p* < 0.05) higher in ApoE-/- rats fed HF than LF. Addition of MB (*p* = 0.104) and MV (*p* > 0.05) had no effects on the ratio.

Mucosal thickness, as measured by the distance between the top of the villi to the muscularis mucosa, was similar between the HF and C groups. The LF group had a tendency towards a lower mucosal thickness in the duodenum than the HF group (Figure 1d, *p* = 0.091). MB and MV groups had a significantly higher mucosal thickness in the duodenum and jejunum (*p* < 0.01 to 0.001) compared to the LF group (Figure 1d,e). In the ileum and colon, there were no differences in mucosal thickness (not shown in any figure).

### 3.3. Monobutyrin and Monovalerin Influence the SCFA Profile in the Blood and Brain

#### 3.3.1. Portal Serum 

The levels of total SCFAs (*p* = 0.07), acetic acid (*p* = 0.058), and isovaleric acid (*p* = 0.065) increased in the portal vein of the MV group compared with the HF group (Table 2). The level of valeric acid and the ratio of acetic-to-butyric acid were significantly higher in the MV group compared with the HF group (*p* < 0.0001 and *p* < 0.05, respectively).

The mean concentration of butyric acid was highest in the MB group, but did not reach significance between the other groups. There was a tendency to a lower ratio of acetic-to-propionic acid in the LF group (*p* = 0.077) compared with the HF group. No changes could be observed for other SCFAs.

#### 3.3.2. Brain

The total amounts of SCFAs were similar in the brain of conventional (C) and ApoE-/- rats (HF) fed the same high-fat diet as well as in ApoE-/- rats fed the low-fat diet (LF). Butyric acid was detected only in one rat in the brain of the HF and LF groups. However, the MB group had a high number of rats with detectable amounts of brain butyric acid (6 rats), followed by the MV group (4 rats). Supplementation of MB therefore significantly increased the amounts of butyric acid in the brain compared with the HF and LF groups (Table 2, *p* < 0.05). The amount of valeric acid (*p* < 0.05) and the ratio of acetic-to-propionic acids (*p* = 0.052) increased in the MB group compared with the LF group, while the amounts of isovaleric acid decreased (*p* < 0.05).

The MV group had lower amounts of propionic acid in the brain compared with the HF and LF groups (*p* = 0.065 and *p* < 0.05, respectively). In contrast, valeric acid increased in the MV group compared with the LF group (*p* < 0.01), and the MV group possessed the lowest amounts of isovaleric acid compared with the LF and HF groups (*p* < 0.01 and *p* < 0.05, respectively). Higher mean values of butyric acid were also found in the MV group when compared with the HF group but did not reach significance (*p* = 0.18).

### 3.4. Monobutyrin and Monovalerin Upregulate the Expression of Tight Junction Proteins and GPR109A Receptor

#### 3.4.1. Small Intestine (Jejunal)

**Figure 2:**
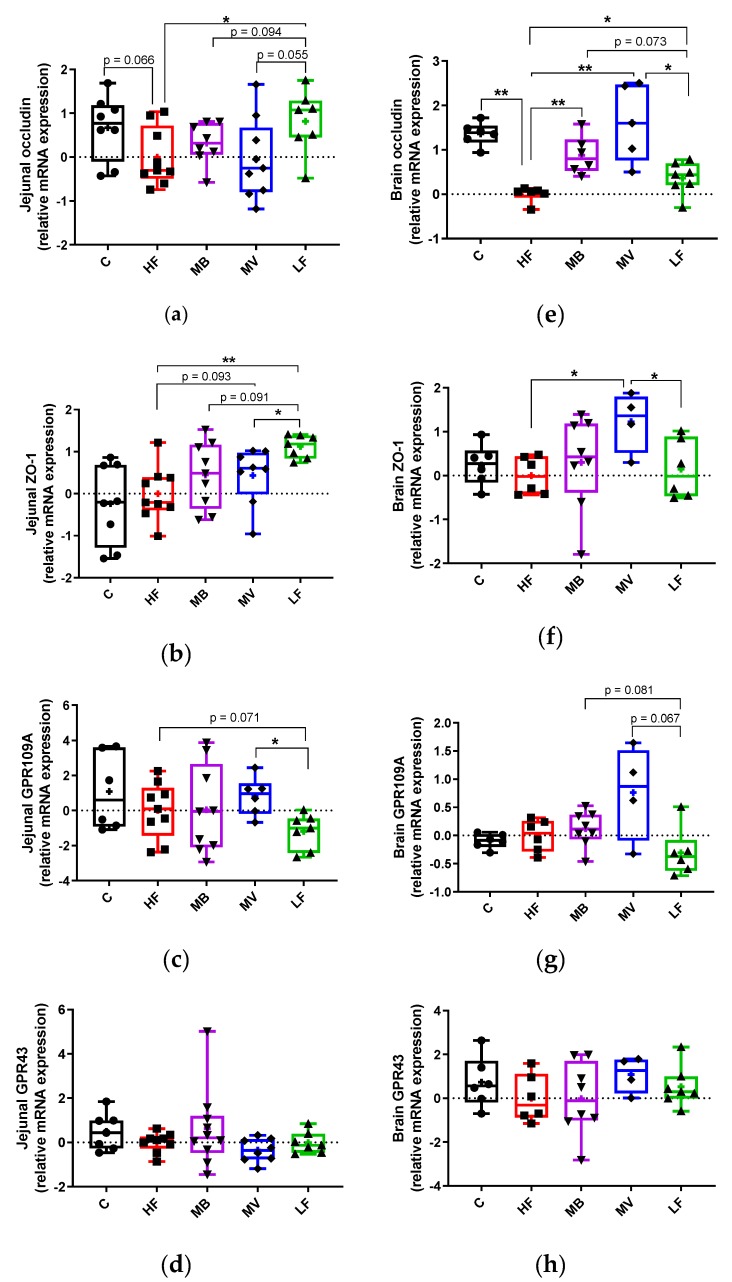
Gene expression of tight junction proteins (zonula occludens (ZO)-1 and occludin) and short-chain fatty acid receptors (G-protein-coupled receptor 43/GPR43 and GPR109A) in the jejunal (**a**–**d**) and brain (**e**–**h**) tissues of conventional rats fed a pure high-fat diet (C), or Apolipoprotein E-knockout (ApoE-/-) rats fed a low-fat diet (LF) or high-fat diet (HF), pure or supplemented with 1% monobutyrin (MB) or monovalerin (MV) for 5 weeks (*n* = 8–10/group). Rats from the C, HF, MB, MV and LF groups are shown as ●, ■, ▼, ♦ and ▲, respectively. Box and whisker plots with error bars indicate minimum and maximum values, while central lines represent median and means shown as “+”. * *p* < 0.05, ** *p* < 0.01.

The expression of occludin was lower in the HF group than in the LF group (Figure 2a, *p* < 0.05) and the HF group also tended to be lower compared to group C (*p* = 0.066). Supplementation with MB and MV to the high-fat diet did not change this fact.

The HF group had significantly (Figure 2b, *p* < 0.01) lower expression of ZO-1 than the LF group. Supplementation with MV increased the expression of ZO-1 to a level between the LF (*p* < 0.05) and the HF (*p* = 0.093) groups, while the increase with the supplementation of MB was insignificant compared with both the LF (*p* = 0.093) and HF (*p* > 0.05) groups. 

GPR109A expression in the HF group was higher than in the LF group (Figure 2c, *p* = 0.071). Including MV in the diet upregulated the expression of GPR109A to a similar level as to that with group C and was significantly (*p* < 0.05) higher than the LF group. 

#### 3.4.2. Brain

The HF group had a lower expression of occludin than the LF group (Figure 2e, *p* < 0.05) and also compared with group C (*p* < 0.01). The expression of occludin increased significantly (*p* < 0.01) in the MB and MV groups. 

The expression of ZO-1 was quite similar in the HF, LF and C groups. The expression of ZO-1 was upregulated in the MV group compared with the HF and LF groups (Figure 2f, *p* < 0.05), while no effects were seen with the MB group.

The expression of the SCFA receptor GPR109A was similar in the HF, LF and C groups. The MB (*p* = 0.081) and MV (*p* = 0.067) groups tended to increase the GPR109A expression compared with the LF group (Figure 2g). The expression of GPR43 was very similar with all groups.

### 3.5. Monobutyrin and Monovalerin Affect Inflammation-Related Biomarkers

**Figure 3:**
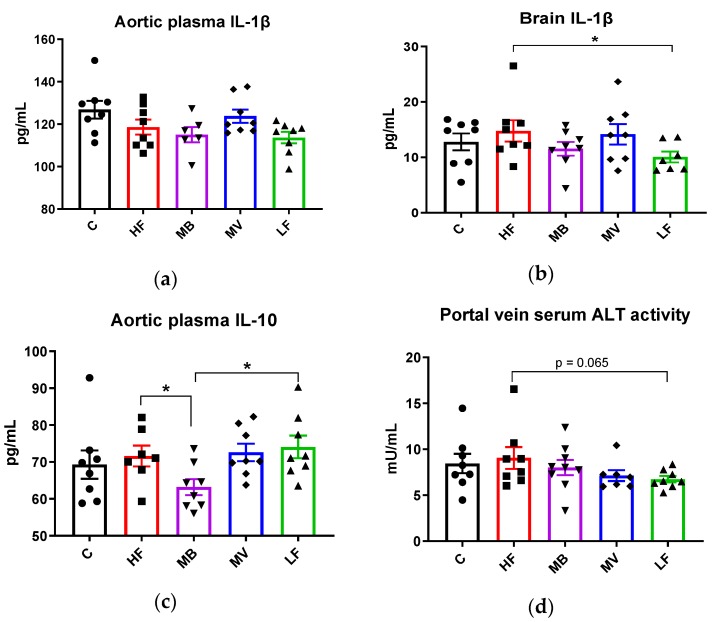
Changes in cytokines and liver enzymes in conventional rats fed a pure high-fat diet (C), or Apolipoprotein E-knockout (ApoE-/-) rats fed a low-fat diet (LF) or high-fat diet (HF), pure or supplemented with 1% monobutyrin (MB) or monovalerin (MV) for 5 weeks (*n* = 8/group). Interleukin-1beta (IL-1β, pg/mL) in (**a**) aortic plasma and (**b**) brain homogenates, (**c**) aortic plasma IL-10 (pg/mL), and (**d**) portal vein serum alanine transaminase activity (ALT) activity (mU/mL). Rats from the C, HF, MB, MV and LF groups are shown as ●, ■, ▼, ♦ and ▲, respectively. Data are presented as means ± SEM. * *p* < 0.05.

ApoE-/- rats fed the high-fat diet (HF group) had similar values of the proinflammatory cytokine IL-1β in the aortic plasma as rats fed the low-fat diet (LF) and also as conventional rats (C group). Adding MB or MV to the high-fat diet gave similar values (Figure 3a). Comparable results were seen for this cytokine in the brain, but the inflammatory marker was higher (Figure 3b, *p* < 0.05) in the ApoE-/- HF group than in the LF group. Adding MB or MV to the diet did not change this. 

The plasma concentration of IL-10 was similar with the HF and the LF groups and also the C group. The MB group had significantly lower plasma concentrations in the aortic blood (Figure 3c, *p* < 0.05) than the HF and LF groups, while the plasma concentrations in the MV group were unaffected.

The HF group had similar ALT activity in the serum portal vein as the C group, but the activity tended to be higher than the LF group (*p* = 0.065). The activity with the MV (*p* = 0.152) and the MB groups (*p* > 0.05) was unaffected. 

Regarding the other parameters, the white blood cell counts tended (Figure S2, *p* = 0.069) to be higher in the HF group than in the C group, while it was very similar compared with the LF group. Neither the MB nor MV groups were affected.

### 3.6. Food Intake, Body and Organ Weights 

**Figure 4:**
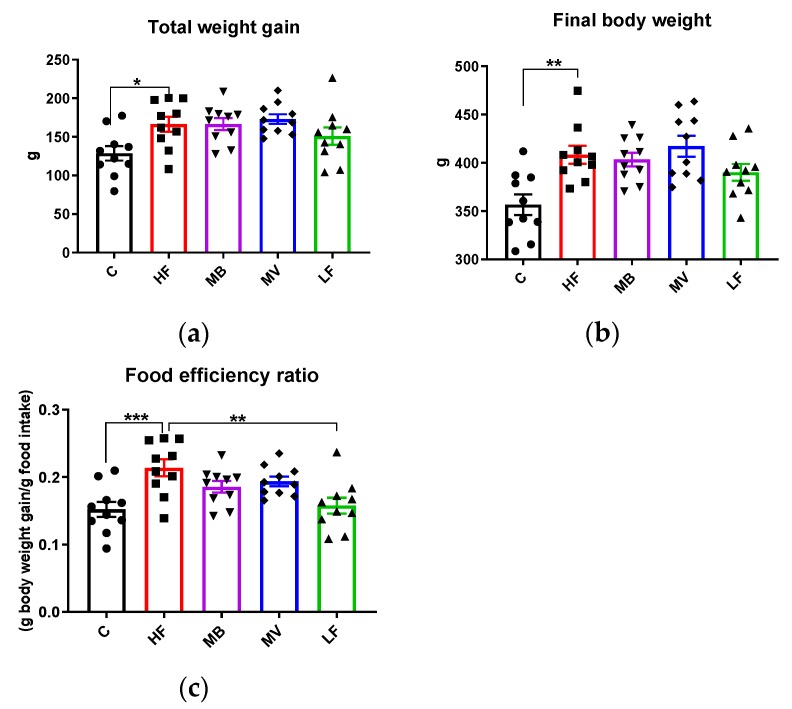
Total weight gain (**a**), final body weight (**b**), and food efficiency ratio (**c**) in conventional rats fed a pure high-fat diet (C), or Apolipoprotein E-knockout (ApoE-/-) rats fed a low-fat diet (LF) or high-fat diet (HF), pure or supplemented with 1% monobutyrin (MB) or monovalerin (MV) for 5 weeks (*n* = 10/group). Rats from the C, HF, MB, MV and LF groups are shown as ●, ■, ▼, ♦ and ▲, respectively. Data are presented as means ± SEM. * *p* < 0.05, ** *p* < 0.01, *** *p* < 0.001.

The HF group had a higher weight gain than the C group (Figure 4a, *p* < 0.05), while it was quite similar to the LF group. MB and MV groups had a similar weight gain as the HF group. Similar results were seen on the final body weight (Figure 4b). The LF group consumed the highest amounts of total food (957 g/rat for 5 weeks), followed by the MB (897), MV (894), C (846), and HF (778) groups. The rats consumed all the food given and no adverse effects could be seen during the entire experiment. The HF group had a higher food efficiency ratio (g body weight gain/g food intake) than the C and LF groups (Figure 4c, *p* < 0.001 and *p* < 0.01, respectively). Most tissue weights, including the liver, spleen, and epididymal, abdominal, and mesenteric fat depots were generally higher in ApoE-/- groups fed the high-fat diets (HF, MB, and MV) than the corresponding values in conventional rats (group C) (Figure S3a–c). The LF group tended (*p* = 0.075) to have a lower caecum weight than the HF group.

Regarding the relative weights, calculated as a percent of the final body weight, brain weight was significantly lower in ApoE-/- rats than in conventional rats (group C, Figure S3b, *p* < 0.05). The relative spleen weight was lower in the HF group than in the LF group (*p* < 0.05). The MV group had a lower (both absolute and relative) value of the caecum weight than the HF group (Figure S3b, *p* < 0.05). 

### 3.7. Multivariate Data Analysis and Correlations 

#### 3.7.1. Multivariate Data Analysis

**Figure 5:**
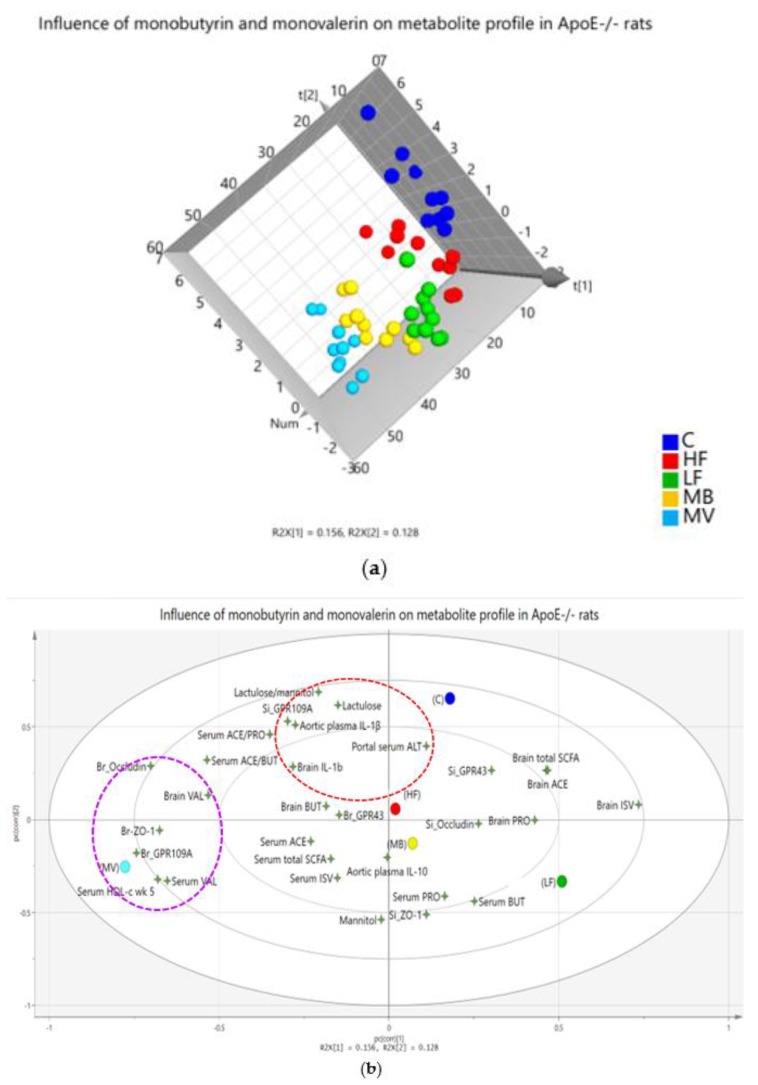
Projection to Latent Structures-Discriminant Analysis (PLS-DA) plot summaries of the effects of monobutyrin (MB) and monovalerin (MV) on short-chain fatty acids (SCFAs) and biomarkers in conventional rats fed a pure high-fat diet (C), or Apolipoprotein E-knockout (ApoE-/-) rats fed a low-fat diet (LF) or high-fat diet, pure (HF) or supplemented with 1% MB or MV for 5 weeks. (**a**) The score scatter plot shows separation patterns among the groups, with each circle denoting one rat having all values of the variables included in the loading plot; (**b**) alterations of the measured variables (shown as 4-angle stars) as an effect of the experimental diets (shown as circles). ACE, acetic acid; BUT, butyric acid; PRO, propionic acid; ISV, isovaleric acid; VAL, valeric acid; ALT, alanine transaminase, IL, interleukin; HDL-c, high-density lipoprotein cholesterol; br, brain; si; small intestine; ZO-1, zonula occludens-1; GPR, G-protein-coupled receptor.

Multivariate data analysis is a useful statistical tool that provides a visual overview of how different variables in complex matrices are connected to each other, i.e., the net effects of all analyses. As seen in this evaluation, there was a comprehensive impact of monobutyrin (MB) and monovalerin (MV) on the SCFA profile (in the portal vein and brain) and the analyzed biomarkers (Figure 5). ApoE-/- rats fed the MB and MV diets were separated from the HF group on the basis of the included metabolites (Figure 5a). Variables responsible for this separation, in descending order, were serum HDL-c, brain GPR109A, serum valeric acid, small intestinal ZO-1, urinary lactulose, brain occludin, brain isovaleric acid, urinary lactulose/mannitol ratio, aortic IL-1β, brain ZO-1, and small intestinal GPR109A, as confirmed by the variable importance for the projection (VIP values > 1 indicate important variables). These determined variables are located on the second and third layers of the ellipse (Figure 5b). 

Supplementation of MB to the high-fat diet drove the effects towards the position of LF (Figure 5b, from left to right of the loading plot), while supplementation of MV was directed in another position far away from the HF group (further down towards the bottom-right side of the loading plot). The MV group is closely associated with a cluster including some markers analyzed in the brain (occludin, ZO-1, and GPR109A) and in serum (HDL-c and valeric acid), which are in the direction (purple dashed ellipse in Figure 5b) opposite to the brain isovaleric acid position. A longer distance between a variable to a group indicates lower values of the variable in this group, and vice versa. This means that brain isovaleric acid was lower, while the other clustered variables were higher with the MV group. Markers associated with intestinal permeability and inflammation (IL-1β, lactulose, lactulose/mannitol ratio, and ALT) are clustered together (red dashed ellipse in Figure 5b) in close proximity to the C and HF groups, indicating that feeding pure high-fat diets to conventional as well as to ApoE-/- rats increased the intestinal permeability and inflammation. In contrast, these effects were lower in the MB, MV, and LF groups, as indicated by the longer distance to these groups. 

#### 3.7.2. Correlations

The correlations between SCFAs and the analyzed biomarkers seem to be dynamic and inter-locational (Figure 5b) and are described in more detail in the Supplementary Materials.

## 4. Discussion

Previous studies have shown that MB and MV can affect lipids as well as SCFA profiles in conventional rats fed high-fat diets. This study shows that the supplementation of MB or MV to high-fat diets also increased serum HDL cholesterol levels, improved the brain SCFA profile, and upregulated the expression of tight junction proteins in ApoE-/- rats fed a high-fat diet. 

### 4.1. Effects of Monovalerin on Lipid Metabolism

It is unclear why feeding high-fat-diets to ApoE-/- rats stimulated HDL-c. Supplementation of MV stimulated the increase of HDL-c concentrations in the portal vein of ApoE-/- rats fed high-fat diets. Similar results were reported with ApoE-/- rats on high-cholesterol or Western diets [26,27]. However, it could be because the high-fat diets have an impact on the gut microbiota, which in turn changes the lipid profile via bile acid metabolism [14,27]. 

In this study, the HDL-c concentration was found to be increased and also positively correlated with portal valeric acid in the MV group. Therefore, valeric acid supplementation may be involved in the increase of HDL-c. Little is known about the role of valeric acid on lipid metabolism, but it has been reported that oral administration of a valeric acid sodium salt suppressed rat liver cholesterol synthesis, independent of a rate-limiting cholesterol synthesis enzyme [28]. An analogue of valeric acid, valproic acid, has been shown to upregulate the expression of genes promoting hepatic uptake of phospholipid and cholesteryl ester from HDL [29] and suppressing increased cholesterol levels in neural stem cells of mice [30]. These findings suggest that MV/valeric acid could contribute to the increase in the HDL-c concentration, but whether it is a positive or negative effect should be clarified in further experiments.

### 4.2. Impact of Monobutyrin and Monovalerin on the Brain SCFA Profile 

Supplementation of 1% MV significantly reduced the amounts of isovaleric acid in the rat brain compared with the ApoE-/- control groups (HF and LF). Similar results were reported in conventional rats fed a high-fat diet supplemented with 0.5% MV [15]. Less is known about the metabolic effects of isovaleric acid; however, fecal isovaleric acid has been correlated with increased depression-associated bacteria and the stress hormone cortisol, a marker for depression, in humans [31]. 

The mechanistic link between isovaleric acid and depression has been addressed to isovaleric acid-mediated inhibition of sodium-potassium adenosine triphosphatase (Na+,K+-ATPase), a protein crucial to preserving the basal potential membrane for normal neurotransmission, in the rat cerebral cortex [32]. The isoform of valeric acid from valerian extracts, isovaltrate, has been shown to act as a potent reverse agonist on adenosine A1 receptors, interfering with the sleep-inducing sedating activities of adenosine [33]. These mechanisms may be best demonstrated in patients with isovaleric acidemia (an inherited metabolic disorder of leucine metabolism caused by isovaleryl-coenzyme A dehydrogenase deficiency), who often suffer from acute encephalopathy. Isovaleric acid has been shown to take part in cholesterol synthesis [34], suggesting a relevant link between lipid alterations and brain disorders. The ability of MV in reducing isovaleric acid suggests it as a promising dietary supplement to prevent isovaleric acid accumulation and associated brain disorders. 

An interesting effect is the accumulation of butyric acid in the brains of ApoE-/- rats fed the MB diet. Elevated levels of butyrate in the brain have been linked with neuroprotective effects against vascular dementia and cerebral ischemia in mice and rats, via an increasing brain-derived neurotrophic factor [35,36,37]. It should be noted that the absence of the ApoE gene compromises the BBB, and the brain uptake of butyrate seems to be quite low (around 0.006% in female baboons) [8,11], but the uptake of butyrate by peripheral organs, such as the spleen and pancreas, is high [8]. Further exploration is justified into whether neuroprotective effects of butyrate are concentration dependent and integrated with other systemic effects, and potential side effects. 

### 4.3. Monobutyrin and Monovalerin Improve the Gut–Brain Barrier Function 

The gene expression data indicated that the supplementation of MB and MV strengthened the BBB of ApoE-/- rats by upregulating expression of the tight junction proteins occludin and ZO-1. These proteins are critical for barrier formation, as reduced levels have been linked to BBB breakdown and a subsequent increasing flux of circulating molecules into the brain that may lead to neurotoxicity and memory impairment in mice [38]. In the white matter of humans with multiple sclerosis, tight junction disruption (ZO-1 and occludin) has been shown to lead to BBB leakage [39]. Tight junction proteins serve as substrates for matrix metalloproteinases (MMPs), whose activity increases after brain injury and in several neurodegenerative disorders. Therefore, the upregulation of ZO-1 and occludin by MB and MV could be partly explained by inhibition of MMP. Butyrate (0.5 and 1.0 mmol/L) has been shown to decrease the activity of MMP-2 and 9 in human fibrosarcoma cell lines [40], and similar effects have been reported for the analogue of valeric acid, valproic acid [41]. BBB leakage in germ-free mice was found to be reinstated after oral administration of sodium butyrate (1 g/kg body weight per day for 3 days) or fecal transfer of the butyrate-producing bacterium *Clostridium tyrobutyricum* [42]. It should be noted that the expression of occludin was negatively associated with brain isovaleric acid, potentially linking BBB integrity, protein breakdown, and the SCFA profile.

### 4.4. Effects of Monobutyrin on IL-10 

The concentration of the cytokine IL-10 was significantly lower in the aortic blood of ApoE-/- rats fed the MB diet than those fed the control diet (HF). IL-10 is an immunomodulatory cytokine with anti-inflammatory properties, but it has been re-evaluated under specific conditions. For example, overexpression of IL-10 has been shown to increase amyloid-β (aβ) accumulation, worsen memory, and increase ApoE expression, especially in the plaque-associated insoluble cellular fraction, while microglial aβ phagocytosis was found to be reduced in amyloid precursor protein (APP) mice, a model of Alzheimer’s disease [43]. These findings correspond with the non-effects of anti-inflammatory strategies for AD patients found in clinical trials [44,45]. 

The effect on IL-10 could be related to inhibition of histone deacetylases (HDACs) by butyrate, which reached the highest levels in both the blood and brain in the MB group. Transcriptional activation of IL-10 is regulated by the suppressor HDAC11 and mediator HDAC6 [46]. Butyrate treatment has been shown to upregulate the expression of both HDAC11 (in bovine cell cultures and seabass liver) [47,48] and HDAC6 (in mouse neuroblastoma and microglia cell cultures) [49]. Due to the involvement of HDAC6 and 11 in mediating IL-10 production, the decrease of IL-10 seems to be associated with butyrate-induced upregulation of HDAC11. The influence of butyrate on IL-10 generation is time specific, as butyrate pre-treatment was found to stimulate IL-10 production at 4 h but then declined at 24 h in human endothelial cells exposed to oxidized LDL [50]. 

## 5. Conclusions

In conclusion, dietary supplementation of MB or MV at 1% to high-fat diets showed increased expression of tight junction proteins in ApoE-/- rats, especially in the brain. These effects were associated with alterations of SCFAs and cytokines. The data suggest MB and MV as potential dietary candidates to prevent metabolic disorders induced by high-fat intake and genetic deficiency. 

## Figures and Tables

**Table 1:** Lipids in the liver and serum from the tail and portal vein in conventional rats fed a pure high-fat diet (C), or in Apolipoprotein E-knockout (ApoE-/-) rats fed a low-fat diet (LF) or high-fat diets (HF), pure or supplemented with 1% monobutyrin (MB) or monovalerin (MV) at week 2, week 4, and at the end (week 5) of the study (*n* = 10/group). Values are means ± SEM.

	C		HF		MB		MV		LF	
	Mean	SEM	Mean	SEM	Mean	SEM	Mean	SEM	Mean	SEM
***Liver Tissue***										
Total CHOL (mg)	527 **	22	692	32	712	42	697	38	685	39
TG (mg)	868 **	88	2503	335	2900	345	2577	356	2490	254
***Tail Vein–week 2***										
Total CHOL (mmol/L)	4 ****	0.2	8	0.4	8 †††	0.4	8 ††	0.4	6 **	0.3
TG (mmol/L)	2.2 **	0.2	3.7	0.2	3.9 †††	0.3	4.0 †††	0.3	2.1 ***	0.1
LDL-c (mmol/L)	3 ****	0.1	4	0.2	4	0.2	5	0.3	3 **	0.1
HDL-c (mmol/L)	2 ****	0.1	4	0.1	4 ††††	0.2	4 ††††	0.2	2 ****	0.1
LDL-c/HDL-c	1.7 *	0.1	1.2	0.1	1.2 †††	0.1	1.2 †††	0.1	1.9 **	0.1
***Tail Vein–week 4***										
Total CHOL (mmol/L)	6 ****	0.2	10	0.5	10	0.2	9	0.3	7 ****	0.3
TG (mmol/L)	1.7 ****	0.1	3.6	0.3	3.3 ††††	0.2	3.4 ††††	0.2	2.0 ****	0.1
LDL-c (mmol/L)	3 ***	0.3	6	0.3	6	0.3	6	0.3	4 *	0.1
HDL-c (mmol/L)	2 ****	0.1	3	0.2	3 †††	0.1	3 ††††	0.2	2 ****	0.1
LDL-c/HDL-c	2.1	0.1	1.8	0.1	2.0	0.1	2.0	0.1	2.1	0.1
***Portal Vein–week 5***										
Total CHOL (mmol/L)	3.9 ****	0.2	7.8	0.4	8.1 †††	0.3	8.7 †††	0.5	5.2 ****	0.1
TG (mmol/L)	2.1 ***	0.2	4.1	0.3	4.2 †	0.2	4.6 †††	0.2	2.7 *	0.2
LDL-c (mmol/L)	2.3 ***	0.1	4.7	0.1	4.8 †	0.3	5.4 ††	0.3	3.2 *	0.1
HDL-c (mmol/L)	1.1 ****	0.1	2.5	0.1	2.5 ††††	0.1	2.9 * ††††	0.1	1.3 ****	0.0
LDL-c/HDL-c	2.1	0.1	2.0	0.2	1.9 †	0.1	1.9 †	0.1	2.4 *	0.1

Group C and LF are significantly different from the ApoE-/- group fed high-fat diet (HF): * *p* < 0.05, ** *p* < 0.01, *** *p* < 0.001, **** *p* < 0.0001. Group MB and MV are significantly different from the ApoE-/- group fed low-fat diet (LF): † *p* < 0.05, †† *p* < 0.01, ††† *p* < 0.001, †††† *p* < 0.0001.CHOL, total cholesterol; TG, triglycerides; LDL-c, low-density lipoprotein cholesterol; HDL-c, high-density lipoprotein cholesterol.

**Table 2:** Short-chain fatty acids in the portal vein and brain in conventional rats fed a pure high-fat diet (C), or Apolipoprotein E-knockout (ApoE-/-) rats fed a low-fat diet (LF) or high-fat diet (HF), pure or supplemented with 1% monobutyrin (MB) or monovalerin (MV) for 5 weeks (*n* = 10/group). Values are means ± SEM.

	C		HF		MB		MV		LF	
	Mean	SEM	Mean	SEM	Mean	SEM	Mean	SEM	Mean	SEM
***Portal vein (µmol/L)***										
Total	595	31	539	43	625	45	647	34	592	66
Acetic acid	487	26	435	35	509	33	527	27	474	54
Propionic acid	39	4	38	6	44	6	41	5	49	6
Butyric acid	33	2	34	4	42	5	30	2	36	4
Valeric acid	3	0.3	4	0.4	3	0.6	15 ****	1.9	4	0.7
Isovaleric acid	14	2	14	3	14	2	23	3	18	2
***Brain (µmol)***										
Total	77	5	73	5	75	5	62	5	74	6
Acetic acid	73	5	69	4	71	5	58	5	70	6
Propionic acid	1.2	0.1	1.3	0.1	1.2	0.1	1.1 †	0.2	1.4	0.1
Butyric acid	0.20	0.10	0.06	0.06	0.32 *†	0.07	0.21	0.09	0.04	0.04
Valeric acid	1.4	0.1	1.7	0.2	1.6 †	0.2	1.6 ††	0.1	1.1 *	0.1
Isovaleric acid	0.9	0.1	0.9	0.1	0.8 †	0.1	0.5 *††	0.1	1.1	0.1

Mean values were significantly different from the HF group: * *p* < 0.05, **** *p* < 0.0001. Mean values were significantly different from the LF group: † *p* < 0.05, †† *p* < 0.01.

## Supplementary Materials

The following are available online at https://www.mdpi.com/2072-6643/12/4/1202/s1, Figure S1: Changes in serum triglycerides measured in the tail vein in individual Apolipoprotein E-knockout (ApoE-/-) rats fed a low-fat diet (LF) or high-fat diets (HF), pure or supplemented with 1% monobutyrin (MB) or monovalerin (MV) for 5 weeks (*n* = 10/group). Figure S2: Other parameters measured in conventional rats fed a pure high-fat diet (C), or Apolipoprotein E-knockout (ApoE-/-) rats fed a low-fat diet (LF) or high-fat diets (HF), pure or supplemented with 1% of monobutyrin (MB) or monovalerin (MV) (*n* = 10/group) for 5 weeks. (a) Portal vein serum glucose (mmol/L), (b) portal vein white blood cell counts. Figure S3: Tissue weights in conventional rats fed a pure high-fat diet (C), or Apolipoprotein E-knockout (ApoE-/-) rats fed a low-fat diet (LF) or high-fat diets (HF), pure or supplemented with 1% monobutyrin (MB) or monovalerin (MV) for 5 weeks (*n* = 10/group). Table S1: Composition of experimental diets (g/kg, dry weight basis) given to conventional rats fed a pure high-fat diet (C) or to Apolipoprotein E-knockout (ApoE-/-) rats fed a low-fat diet (LF) or high-fat diets (HF), pure or supplemented with 1% monobutyrin (MB) or monovalerin (MV) for 5 weeks.
