# Supplementary material for: Monobutyrin and Monovalerin Affect Brain Short-Chain Fatty Acid Profiles and Tight-Junction Protein Expression in ApoE-Knockout Rats Fed High-Fat Diets
Source: Nutrients. 2020 Apr 24;12(4):1202. doi: 10.3390/nu12041202 (PMC7230324; doi:10.3390/nu12041202)
Supplement: Supplementary file 1 [file nutrients-12-01202-s001.pdf]

Supplementary material

Materials and Methods

Supplementary Table S1. Composition of experimental diets (g/kg, dry weight basis) given to conventional rats fed a pure high-fat diet (C) or to Apolipoprotein E-knockout ($ApoE^{-/-}$) rats fed a low-fat diet (LF) or high-fat diets (HF), pure or supplemented with 1% monobutyrin (MB) or monovalerin (MV) for 5 weeks. .

Ingredients/groups	C	HF	LF	MB	MV
**Macronutrients**					
Protein					
Casein†	150.0	150.0	150.0	150.0	150.0
DL-Methionine†	1.2	1.2	1.2	1.2	1.2
Fat					
Lard	230.0	230.0	-	230.0	230.0
Rapeseed oil	-	-	50.0	-	-
Carbohydrate					
Wheat starch¶	410.8	410.8	590.8	400.8	400.8
Sucrose	100.0	100.0	100.0	100.0	100.0
Cellulose§	50.0	50.0	50.0	50.0	50.0
**Micronutrients**					
Mineral mixture‖	48.0	48.0	48.0	48.0	48.0
Vitamin mixture‖	8.0	8.0	8.0	8.0	8.0
Choline chloride†	2.0	2.0	2.0	2.0	2.0
**Supplements**					
Monobutyrin	-	-	-	10.0	-
Monovalerin	-	-	-	-	10.0

MB, monobutyrin; MV, monovalerin.

† Sigma-Aldrich, St. Louis, MO, USA

§ FMC BioPolymer, Cork, Ireland

‖ Altromin, Lage, Germany, with composition according to the American Institute of Nutrition

¶ Cargill, Sas van Gent, The Netherlands; the amount varied depending on the supplement and fat content of the test diets

Results

a)

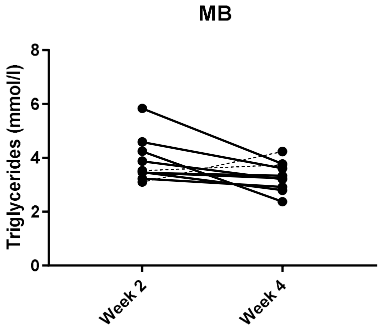

b)

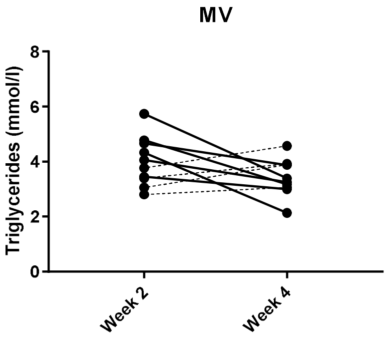

c)

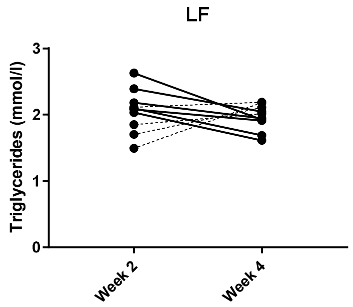

d)

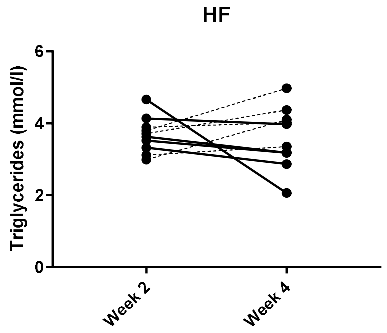

Figure S1. Changes in serum triglycerides measured in the tail vein in individual Apolipoprotein E-knockout ($ApoE^{-/-}$) rats fed a low-fat diet (LF) or high-fat diets (HF), pure or supplemented with 1% monobutyrin (MB) or monovalerin (MV) for 5 weeks (n = 10/group). Solid lines indicate decreased changes in triglycerides between week 2 and 4, while dash lines show increased changes. Values are means ± SEM. * $p < 0.05$, **** $p < 0.0001$.

a) b)

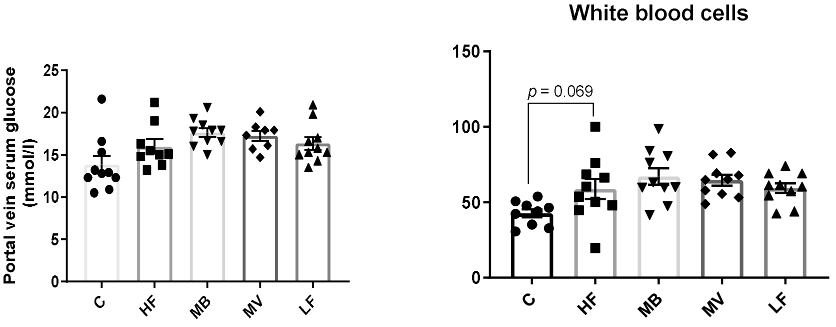

Figure S2. Other parameters measured in conventional rats fed a pure high-fat diet (C), or Apolipoprotein E-knockout (ApoE$^{-/-}$) rats fed a low-fat diet (LF) or high-fat diets (HF), pure or supplemented with 1% of monobutyrin (MB) or monovalerin (MV) (n = 10/group) for 5 weeks. (**a**) Portal vein serum glucose (mmol/L), (**b)** portal vein white blood cell counts.

a)

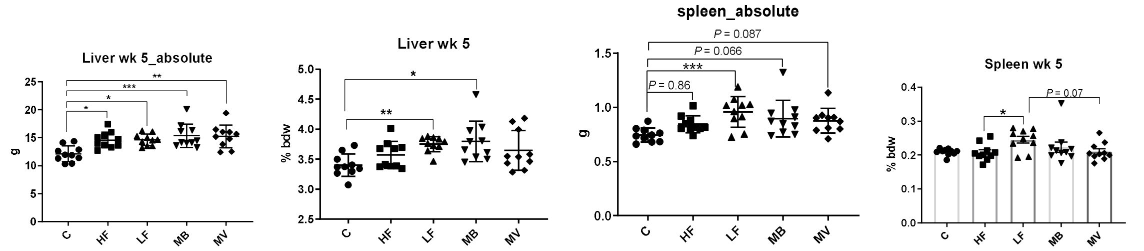

b)

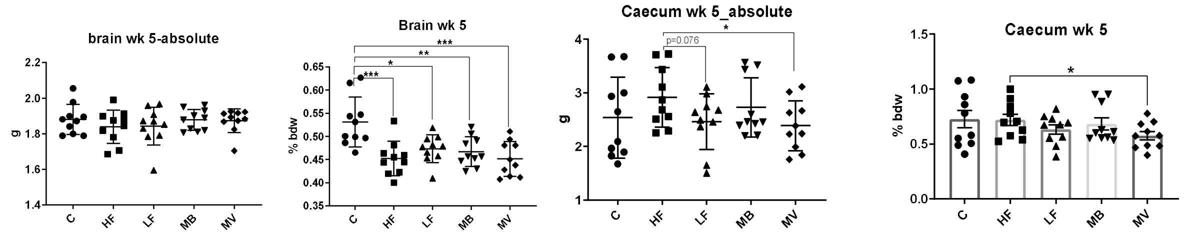

c)

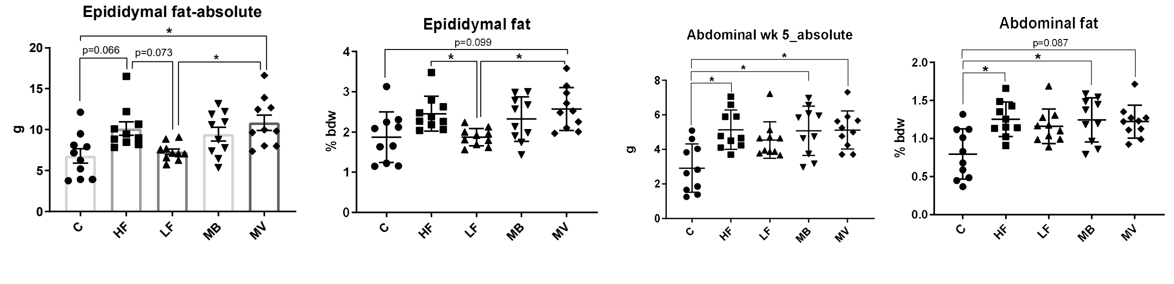

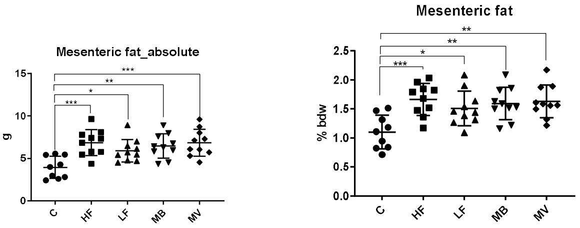

Figure S3. Tissue weights in conventional rats fed a pure high-fat diet (C), or Apolipoprotein E-knockout ($ApoE^{-/-}$) rats fed a low-fat diet (LF) or high-fat diets (HF), pure or supplemented with 1% monobutyrin (MB) or monovalerin (MV) for 5 weeks (n = 10/group). Significant differences between groups: * $p < 0.05$, ** $p < 0.01$, *** $p < 0.001$.

Correlations

Correlations between SCFA and the analysed biomarkers seem to be dynamic and inter-locational (**Figure 5b** in the manuscript). For instance, in serum, valeric acid, highest in the MV group, was positively associated with acetic and isovaleric acids (p = 0.011 to 0.002, r = 0.39 to 0.46), but there was no correlation with butyric acid. In the brain, valeric acid was positively correlated with butyric acid (P = 0.002, r = 0.48). Isovaleric acid was positively associated with total SCFA ($p < 0.0001$, r = 0.57), acetic acid ($p < 0.0001$, r = 0.56), and propionic acid ($p < 0.0001$, r = 0.78), but negatively related to valeric acid (p = 0.049, r = -0.32). There were also some correlations between serum and brain; serum valeric acid was conversely correlated with brain butyric (p = 0.046, r = -0.34) and isovaleric acids (p = 0.017, r = -0.39).

In the brain, mRNA expression levels of ZO-1 and GPR109A were positively correlated with both butyric (p <0.0001, r = 0.78 and p = 0.0005, r = 0.72) and valeric acids (p = 0.001, r =0.68 and $p < 0.0001$, r = 0.78), while brain occludin was negatively correlated with only brain isovaleric acid (p = 0.050, r = -0.43).

Aortic plasma IL-1β showed a positive association with brain valeric acid (p = 0.025, r = 0.43), while brain IL-1β was negatively correlated with serum propionic (p = 0.001, r = -0.54) and butyric acids (p = 0.019, r = -0.38) and positively associated with serum ratio of acetic-to-propionic acid (p = 0.0005, r = 0.053).

Serum HDL-c measured at week 5 was positively correlated with valeric acid in blood (p = 0.0046, r = 0.41) and brain (p = 0.0006, r = 0.53). HDL-c was positively correlated with brain expression of GPR109A

(*p* = 0.006, r = 0.49) and ZO-1 (*p* = 0.026, r = 0.41), and inversely related with jejunal expression of occludin (*p* = 0.04, r = -0.32).

Lactulose in urine was positively associated with serum acetic acid (*p* = 0.032, r = 0.306) and negatively associated with expression of jejunal ZO-1 (*p* = 0.010, r = -0.40).

Expression of GPR109A in the jejunum was negatively correlated with serum levels of butyric (*p* = 0.003, r = -0.47) and propionic (*p* = 0.028, r = -0.36) acids, so positively associated with the ratios of acetic-to-butyric acids (*p* = 0.004, r = 0.46) and acetic-to-propionic acids (*p* = 0.015, r = 0.40).
